# Heat stress on calves and heifers: a review

**DOI:** 10.1186/s40104-020-00485-8

**Published:** 2020-08-10

**Authors:** Jingjun Wang, Jinghui Li, Fengxia Wang, Jianxin Xiao, Yajing Wang, Hongjian Yang, Shengli Li, Zhijun Cao

**Affiliations:** 1 State Key Laboratory of Animal Nutrition, Beijing Engineering Technology Research Center of Raw Milk Quality and Safety Control, College of Animal Science and Technology, China Agricultural University, Beijing, 100193 PR China; 2 Department of Animal Science, University of California, Davis, California 95616 USA; 3 Beijing CNAgri Animal Science Technology Research Center, Beijing, 100193 PR China

**Keywords:** Calf, Energy, Heat stress, Heifer, Reproduction

## Abstract

The current review is designed with aims to highlight the impact of heat stress (HS) on calves and heifers and to suggest methods for HS alleviation. HS occurs in animals when heat gain from environment and metabolism surpasses heat loss by radiation, convection, evaporation and conduction. Although calves and heifers are comparatively heat resistant due to less production of metabolic heat and more heat dissipation efficiency, they still suffer from HS to some degree. Dry matter intake and growth performance of calves and heifers are reduced during HS because of redistributing energy to heat regulation through a series of physiological and metabolic responses, such as elevated blood insulin and protein catabolism. Enhanced respiration rate and panting during HS accelerate the loss of CO_2_, resulting in altered blood acid-base chemistry and respiratory alkalosis. HS-induced alteration in rumen motility and microbiota affects the feed digestibility and rumen fermentation. Decreased luteinizing hormone, estradiol and gonadotrophins due to HS disturb the normal estrus cyclicity, depress follicular development, hence the drop in conception rate. Prenatal HS not only suppresses the embryonic development by the impaired placenta, which results in hypoxia and malnutrition, but also retards the growth, immunity and future milk production of newborn calves. Based on the above challenges, we attempted to describe the possible impacts of HS on growth, health, digestibility and reproduction of calves and heifers. Likewise, we also proposed three primary strategies for ameliorating HS consequences. Genetic development and reproductive measures, such as gene selection and embryo transfers, are more likely long-term approaches to enhance heat tolerance. While physical modification of the environment, such as shades and sprinkle systems, is the most common and easily implemented measure to alleviate HS. Additionally, nutritional management is another key approach which could help calves and heifers maintain homeostasis and prevent nutrient deficiencies because of HS.

## Introduction

Livestock could maintain a constant body temperature within a specific environmental temperature range, known as the thermoneutral zone (TNZ), and achieve minimal physiological costs and maximal productivity [1]. The TNZ of a 1 month old calf is between 13 to 25 °C, and the TNZ of a heifer with 0.8 kg daily gain is between 0 to 15 °C [2]. Heat stress (HS) occurs when the body temperature of the livestock increases and they cannot dissipate body heat adequately to maintain thermal equilibrium, which is due to an elevated ambient temperature above TNZ along with high humidity and slow air movement [3, 4]. This heat accumulation would result in compromised performance and reproduction and increased mortality of livestock. It was estimated that total annual economic losses to livestock industries caused by HS amounted to between $1.69 and $2.36 billion across the United States [5]. Thus, HS is a significant challenge in the animal husbandry.

Most studies on the effect of HS delve on production inefficiencies like reduced milk yield and death in mature dairy and beef cattle [6–8]. Besides, calves and heifers generate less metabolic heat and have greater body surface area relative to body mass, hence efficiently dissipating body heat and are thus considered to be more tolerant of HS than mature cattle [9]. However, scientific evidence has shown influences of HS on the physiology, feed conversion efficiency, rumen and reproduction of calves and heifers [10, 11]. These impacts play an essential role in their future productive life. Therefore, attention on calves and heifers under HS is required. A clear understanding of HS effect on calves and heifers is necessary to help develop strategies to mitigate the related adverse effect. Physical modification of the environment, genetic development and reproductive measures, and nutritional management seem to be three major practices [12] to help calves and heifers respond to the challenge of HS. Keeping in view the emerging issue of HS, the current review is designed with objectives to assess the impact of HS on calves and heifers and attempt to highlight the practical methods for HS alleviation.

### Effect of heat stress on calves and heifers

#### The indicator of heat stress level

Typically, the level of HS in cattle is estimated by using temperature-humidity index (THI), which was first introduced by Thom [13]. This index has been adapted to describe ambient temperature and humidity that cause HS on cattle [14]. Segnalini et al. [15] have divided THI into six categories defining the level of HS on dairy cattle. However, THI values only serves as a rough measure of HS effect on production [16]; and they call for necessary adjustments, because the environmental stimulus includes other factors, such as wind speed and solar radiation [8]. Moreover, the THI threshold for calves and heifers remains unknown because of very limited information available related to THI and HS on calves and heifers. More studies will help to quantify THI for calves and heifers and even explore new indices to indicate the level of HS.

#### Heat stress effect on dry matter intake, growth performance, mortality and water intake

HS exerts a negative effect on the dry matter intake (DMI) and growth performance of calves and heifers. It was reported that dairy calves born in summer tended to have lower average daily gain (ADG) than those born in winter [17]. Because calves consume a given volume of milk or milk replacer and starter *ad libitum* daily, the main effect of HS on DMI for calves might lie in the starter. Rauba et al. [18] reported that calves born in summer had lower starter DMI than those born in winter. Broucek et al. [19] showed that calves under HS conditions (74.8 of THI) had reduced starter intake compared with those raised under moderate conditions (59.7 of THI). Colditz and Kellaway [10] showed that heifers raised under HS condition (38 °C environment) had reduced feed intake and ADG compared to those maintained under cool ambient conditions (17 °C environment). Similarly, Baccari et al. [20] also reported lower feed intake, ADG and feed efficiency of Holstein heifers under HS conditions (32.5 to 34 °C environment) compared with cooler conditions (18 to 20 °C environment). Moreover, Nonaka et al. [21] found that daily dry matter intake and ADG of prepubertal Holstein heifers at 33 °C environment dropped by 9% and 22%, respectively, compared to those raised at 28 °C environment, while water intake increased by 23% due to additional evaporative water loss, such as sweating. In addition to these findings, a study quantified the adverse effect of HS on the productivity of dairy replacements under 2 years of age. In this study, the responses of dairy replacements under 2 years of age to HS, such as decreased DMI losses, daily gain and increased monthly mortality, were modeled from literature data with equations using a combination of maximum THI, daily duration of HS, and a heat load index. Then this study estimated the economic losses from HS based on these models, and the number was $48 million per year [5].

#### Heat stress effect on heat production and energy allocation

**Fig. 1:**
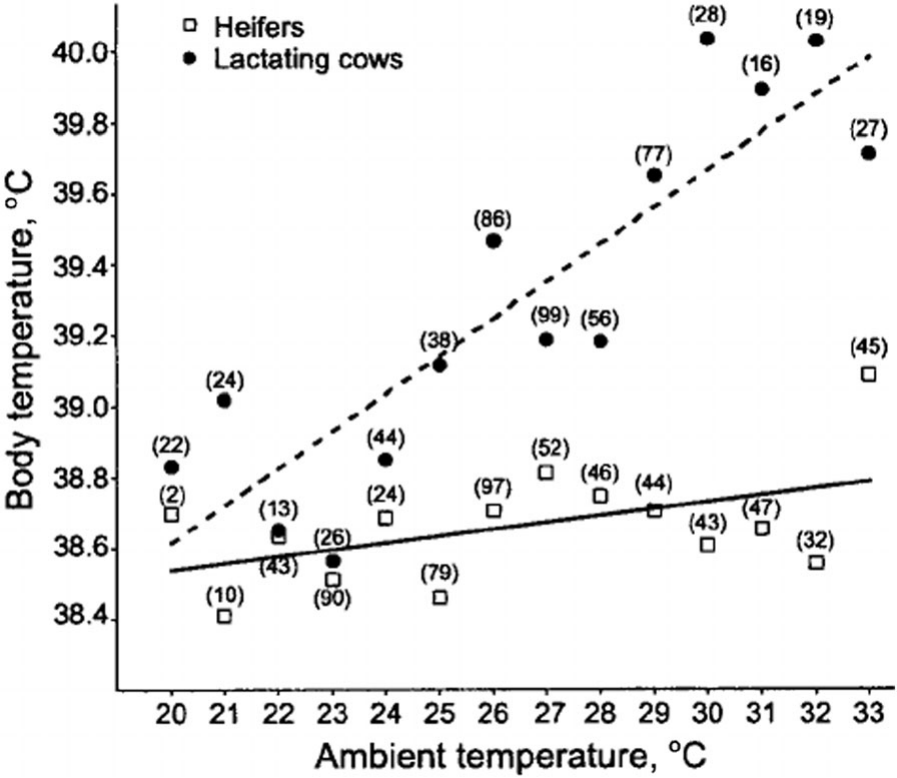
Relationship between ambient temperature (AT) and body temperature (BT) in lactating cows and nulliparous heifers. Values within parentheses represent the number of observations of BT for each group evaluated for each degree of AT. Calculated linear regression for cows was BT = 0.11AT + 36.49 (dashed line) and for heifers was BT = 0.02AT + 38.05 (solid line). Adapted from Sartori et al. [23].

During HS, heat gain of calves and heifers from environment and metabolic processes would exceed heat loss through radiation, convection, evaporation and conduction. Thus heat accumulates in calves and heifers, resulting in a rise in body core temperature. It has been reported that heat production and rectal temperature of heifers at 30 °C environment were significantly higher than those at 15 °C environment [22]. Similar results of calves and heifers under HS conditions (around 32.5 to 34 °C environment) were observed by several other researchers [20, 21]. Sartori et al. [23] pointed out the positive relationship between ambient temperature and body temperature in nulliparous dairy heifers (11 to 17 months old) by establishing linear regression equation, which not only indicated the change in body temperature for heifers during HS but also suggested that heifers were more tolerant of HS than lactating cows because of the less changes in body temperature (Fig. 1). Calves and heifers would reduce heat gain and elevate heat loss to adapt to HS. Once they fail to adjust to the significant heat gain, they might end up dying [1]. Reduced feed intake, as previously mentioned, might contribute to the decline of heat gain from digestion and metabolism [24]. Besides, long standing time of heifers under HS conditions (40 °C environment for 4 h daily) was reported [25], which was supposed to expose more surface area for heat abatements, such as sensible heat loss and convection via air movement [26].

As environmental temperature rises, the energy intake of calves and heifers decreases because of compromised DMI while the energy expenditure on maintenance and metabolism increases to remove the heat load. Consequently, calves and heifers under HS would sacrifice a fraction of growth energy for heat regulation, which could partly explain the decreased growth performance and productive efficiency [21, 22]. Besides, it has been reported that prepubertal heifers (around 7.7 months old) under HS conditions (33 °C environment) might change the way of storing body energy, such as reduced protein retention and thicker fat [21]. The mechanism for this remains unknown. More studies are required to explain this way of storing body energy.

#### Heat stress effect on physiology

Considerable physiological changes take place in blood flow, acid-base chemistry and hormones, as the responses of calves and heifers to HS. Located throughout the bodies of cattle, neurons are temperature sensitive and send information to the hypothalamus, which produces numerous physiological and behavioral responses in the attempt to maintain heat balance.

It has been reported that heifers experience an increased heart rate during HS [22, 25]. This helps maintain blood pressure as a response to the elevated vasodilation and increased blood flow caused by HS [22]. Moreover, blood flow is redistributed to peripheral tissues, which ensures body heat is transported from organs to the body surface [27]. Thus, the change in heart rate could serve as a protective mechanism of heifers to maintain their heat balance.

Increased respiration rate and panting were reported in nulliparous heifers under HS [21, 22]. This ensures that the excess heat is dissipated through enhanced evaporative cooling [2], resulting in an altered blood acid-base chemistry [9]. For lactating cows, enhanced respiration rate and panting lead to greater loss of CO_2_ via pulmonary ventilation, reduced blood concentration of carbonic acid and upsetting the critical ratio of carbonic acid to bicarbonate necessary to maintain blood pH, eventually a respiratory alkalosis [28]. Therefore, they need to compensate for higher blood pH by increasing urinary bicarbonate excretion with the loss of blood carbonic acid [29]. Moreover, lactating cows under HS exhibited a variation in blood pH and bicarbonate levels, which was relative to their respiratory rate [30]. Though researches in calves and heifers are lacking, it is most likely that the response of calves and heifers to respiratory alkalosis would be similar to that of lactating cows, because of their reported increased respiration rate and panting.

HS could lead to hormonal alterations. For calves and heifers under HS, increased insulin and decreased thyroid hormones were frequently reported [20, 21, 31, 32]. Although the contribution of hormones to HS requires more studies, there is a plausible explanation from cellular and tissue standpoints. Several studies have revealed that the exposure to heat could enhance cellular reactive oxygen species (ROS) production and induces oxidative stress, which leads to the cytotoxicity [33]. The disturbance of the steady state level of ROS production induces the inactivation of the respiratory chain, then mitochondria are damaged and cells fail to meet enhanced energy requirements and the demand for more metabolic substrates increases, such as glucose [34]. Thus, glucose uptake by tissues or organs is stimulated through the escalation of insulin, but central nervous and immune systems, which are obligate glucose users [35], would take a high priority of adequate glucose supply over other tissues. This change in the hierarchy of glucose utilization decreases the allocation of glucose to the mammary gland and skeletal muscles [36]. Additionally, insulin prevents calves and heifers under HS from mobilizing adipose tissue [37]. This might lead to an accelerated protein catabolism in the mammary gland and muscles for more energy substrate [36], and compromised growth performance, which also might be related with reduced thyroid hormone for its positive correlation with weight gain and tissue development [38]. These alterations in physiology and following energy metabolism afterwards tend to be adaptive mechanisms employed to prioritize the maintenance and are responsible for reduced growth performance during HS. More deep knowledge is still warranted to take an insight into the mechanism of how HS influences the growth performance of calves and heifers.

#### Heat stress effect on rumen motility, microbiota and fermentation

In response to HS, heifers tend to reduce the gastrointestinal motility to decrease metabolic heat, hence a reduction in the passage rate of digesta [30]. Several researchers have reported a lower ruminal passage rate of feed in heifers under HS conditions. Nonka et al. [21] reported that prepubertal heifers at 33 °C had 56% lower ruminal passage rate compared to that of heifers at 20 °C. Bernabucci et al. [39] also showed that significant difference in ruminal passage of 10 months old heifers under 84 and 64 of THI. Increased retention time in the whole gastrointestinal tract, along with the reduced DMI, would lead to higher digestibility in heifers under HS conditions [40]. Such an increment in digestibility was indicated by previous studies [10, 21, 39, 41]. However, some contrasting results were available regrading decreased digestibility of cattle under HS conditions (32.2 °C environment) [42]. This reduction could be explained by some factors, such as the dilution of rumen contents caused by increased water intake [43], and lower rumen and intestinal absorption of nutrients due to reduced blood flow [44]. Furthermore, Bernabucci et al. [39] suggested that these changes in digestibility were not only because of the passage rate and DMI but also due to other factors mentioned before, which might serve as an adaptive response of the digestive tract to HS.

Besides, rumen microbiota and fermentation of heifers would change during HS. During HS, the community of ruminal microbiota is significantly restructured due to the alteration in the composition and volume of feed, leading to the change of the ruminal fermentation product [45, 46]. Uyeno et al. [47] showed that the relative abundance of the *Clostridium coccoides–Eubacterium rectale* group, which is a cluster of butyrate-producing bacteria [48], and the genus *Streptococcus* increased while the genus *Fibrobacter*, a representative of acetate producing bacteria [49], decreased in heifers under HS conditions (33 °C environment). Thus, some researches reported that heifers under HS conditions (around 32 to 33 °C environment) had decreased amount of volatile fatty acids (VFAs), reduced amount and concentration of acetic acid and increased amount and concentration of butyric acid [21, 41, 50]. These changes might contribute to the impaired growth performance of growing cattle because VFAs serve as primary energy supply of them and the amount they could utilize reduced [41]. More in-depth studies are suggested to explore the dynamics of ruminal microbiota and how this alteration might affect rumen fermentation and performance of heifers under HS.

#### Heat stress effect on reproduction

HS not only affects the whole reproduction stage of heifers from estrous to calving period, but also generates a lasting effect on newborn calves. The duration and intensity of estrus are reduced by HS [51, 52], resulting in the silent heat or weak estrus expression thus the difficulty in breeding [53]. Besides, HS might depress follicular development [54] by impairing follicle selection, delaying follicular wave, reducing follicular dominance and compromising follicular steroidogenesis, eventually leading to the poor quality of oocytes [55–59]. And the possibility of successful inseminations might be curtailed by HS because of altering the intrauterine environment [60]. Therefore, conception rate drastically drops during HS. The physiological mechanism regulating the effect of HS on reproduction is still unclear. However, the hormone regulatory axis might serve as a probable explanation [24, 61]. HS could increase the adrenocorticotropic hormone (ACTH) secretion, which was reported to block estradiol-induced sexual behavior [62], and the cortisol secretion, which could inhibit the gonadotropin-releasing hormone (GnRH) and luteinizing hormone (LH) secretion and affect the hypothalamic-hypophyseal-ovarian axis [63]. These increased secretions could lead to hindered luteolysis, altered follicle dominance and disrupted ovulation [63]. Though most of related researches focused on mature cows, these discussions below might fit for both breeding heifers and cows.

HS also suppresses the embryonic development [64] and produces a carryover effect on newborn calves [65]. Although the effect of HS on embryos is different at various developmental stages [66], the survival of early embryos in first 7 d is particularly susceptible to HS for disturbing heat shock protein synthesis [67], oxidative cell damage [68], impaired interferon-tau production for pregnancy maintenance [54] and the expression of genes related with apoptosis [69]. As the pregnancy proceeds, the effect of HS on embryonic survival diminishes [70]. Because the fetus grows at the fastest rate and gains 60% of its birth weight during the last 2 months of gestation [71], HS during this period could lead to fetal growth retardation and compromised postnatal growth [65]. Due to HS-induced low uterine and umbilical blood flow [72], placental development is depressed, represented as reduced placental weight [73] and circulating placental hormones [74]. Fetus relies on the placenta for the supply of oxygen and nutrients [75]. Thus impaired placental development could cause a hypoxic state and compromised intake of nutrients, such as glucose and amino acids [65]. This insufficient placental supply would consequently retard the growth of the fetus and have profound effects on postnatal growth [76]. Available researches have revealed that the birth weight and growth rate of calves born from late gestation heat stressed cows were low [77–79]. In addition to the growth, prenatal HS might exert a negative influence on the immunity of newborn calves. HS might cause the alteration in colostrum composition, such as lower Ig content [80], and impaired passive immune transfer because of decreased enterocyte turnover in the small intestine [81]. Besides, prenatal HS influences the maternal environment and might induce epigenetic modifications in the development of normal fatal immune cells and hence depressing the cell-mediated immune function in neonatal calves [77]. Moreover, negative prenatal HS effects could persist in the future stage of calves. Monteiro et al. [82] reported that calves experiencing maternal HS produced less milk in the first lactation than calves with maternal cooling environment. More studies are necessary to elucidate the effects of prenatal HS on the fetus and neonatal calves and the mechanism underlying it.

### Strategies to alleviate heat stress

Factors that influence the extent of HS on calves and heifers include genetic, reproductive, environmental and nutritional aspects. Strategies developed based on these aspects could ameliorate HS on calves and heifers.

#### Genetic development and reproductive measures

Breed plays an important role in genetic influence on the HS tolerance of calves and heifers, thus there appears to be benefits from hybrid vigor under HS conditions [10] and crossing Holstein cows with domestic dairy breeds would potentially enhance the HS tolerance. However, whether these cross-breeds are sufficiently productive to meet the needs of the dairy industry remains questionable. Generally, dairy cows seem to be more sensitive to HS as milk production elevates metabolic heat production. Therefore, breeding dairy cows selectively for milk yield would increase their susceptibility to HS [54]. But selecting particular genes that control traits related to thermotolerance would be desirable as the only thermal resistance would be selected for without compromising milk yield [83]. The slick hair gene, which results in a short, sleek and glossy hair coat, has been introduced to improve the thermoregulatory ability because the hair color is associated with solar radiation absorption and the hair length is related to convective and conductive heat loss [84]. Heat shock genes could also serve as markers in marker-assisted selection for thermotolerance in that heat shock protein protects cells from HS by maintaining cellular machinery and cellular apoptosis [85, 86]. However, it might not be practical only to select heat shock genes, some target traits should be set together to lead the specific genetic development. Besides, embryo transfers at 7 to 8 d after estrus, which is the most thermosensitive period of survival, could bypass the effect of HS on early embryos and increase the pregnancy rate [87, 88]. The addition of survival factors to bovine embryos, such as antioxidants and insulin-like growth factor-1, has been reported to minimize the inhibition of embryonic development and apoptosis induced by HS and thus improve embryo HS resistance [89, 90].

#### Physical modification of the environment

Modification of the environment could reduce the heat gain and elevate the heat dissipation to protect calves and heifers from HS. The most prevalent measures to alleviate HS are the provision of house or shade (together with feed and water), evaporative cooling with water in the form of fog, mist, or sprinkling with natural or forced air movement, as well as cooling ponds [91]. Water sprinkling with ventilation could enhance evaporation, which serves as the dominant mode of heat dissipation for heifers under HS conditions [92]. Exposed to HS conditions (36 °C environment), heifers sprayed with water were reported to have a lower rectal temperature, respiratory rate and 26.1% higher weight gain compared with non-cooled heifers [93]. Similarly, with the use of fans, the ADG and feed efficiency of dairy calves increased significantly by 23% and 21% respectively under HS conditions (around 29 °C environment) [94]. Moreover, Moghaddam et al. [95] reported with sprinkler and fan, the cooling of dairy heifers for a short time before and after artificial insemination could increase the pregnancy rate during HS (36.1 °C environment). In addition, shading is regarded as one of the most easily implemented and cost-effective methods to minimize heat load from solar radiation. Well-designed shade could reduce 30% to 50% head load from solar radiation [96]. Marcillac-Embertson et al. [97] reported that in corrals, heifers with 65.0 m^2^ shade had significantly higher DMI and ADG than sprinkle cooled heifers with 5 times daily from 11:00 to 19:00 h and 7 min of each period. Under this housing system and cooling ways, shade might be more effective than sprinkle systems for heifers during HS.

#### Nutritional management

Nutritional management could help calves and heifers to maintain homeostasis or prevent nutrient deficiencies because of HS. The starter intake of calves might be depressed by HS [94], leaving them with less energy available to support high energy requirements. The nutritional management of liquid feed for calves, such as intensity and nutrient density, should be considered to support high energy requirements based on different situation. Like mature cattle, the nutrient density of the ration should be raised to overcome the decline in DMI of heifers [9]. Given that greater heat production is associated with the metabolism of acetate compared with propionate in rumen [98], it would be reasonable to feed heifers more concentrates at the expense of fibrous ingredients to increase the nutrient density and decrease the heat increment [99]. This method, however, should take into consideration the need of heifers for adequate fiber to ruminate and maintain health. Besides, the addition of dietary fat could also be advantageous to heifers, because this has improved the efficiency of the conversion of dietary fat to body fat and lowered the heat increment compared to protein and carbohydrates [99]. The supplementation of vitamins A, C and E and mineral, such as zinc, could relieve the oxidative damage due to HS, and the regulation of feed electrolyte by dietary cation-anion difference (DCAD) could help maintain the blood acid-base balance and correct the mineral deficiency of Na and K due to sweating during HS [99–101].

## Conclusion

Based on the available information in literature, we concluded that although calves and heifers are supposed to be more tolerant of HS than mature cattle, they still suffer from HS to some degree. In order to acclimatize to HS, calves and heifers experience a series of physiological and metabolic changes to achieve the redistribution of energy, hence the compromised growth performance. HS-induced alteration in rumen motility and microbiota leads to the change of feed digestibility and production of rumen fermentation. HS reduces the duration and intensity of estrus, depresses follicular development by the regulation of reproduction hormone and suppresses the embryonic development by impaired placenta and the growth and immunity of the offspring. Genetic development and reproductive measures, physical modification of the environment and nutritional management are three major strategies to ameliorate HS. Productive benefits should be taken into consideration when developing strategies to ameliorate HS.

## Data Availability

Not applicable.

## Abbreviations

ACTH: Adrenocorticotropic hormone
ADG: Average daily gain
AT: Ambient temperature
BT: Body temperature
DCAD: Dietary cation-anion difference
DMI: Dry matter intake
GnRH: Gonadotropin-releasing hormone
HS: Heat stress
LH: Luteinizing hormone
ROS: Reactive oxygen species
TNZ: Thermoneutral zone
THI: Temperature-humidity index
VFAs: Volatile fatty acids

## References

[1] Kadzere CT, Murphy MR, Silanikove N, Maltz E (2002). Heat stress in lactating dairy cows: a review. Livest Prod Sci.

[2] Hahn GL (1997). Dynamic responses of cattle to thermal heat loads. J Anim Sci.

[3] Morrison SR (1983). Ruminant heat stress: effect on production and means of alleviation. J Anim Sci.

[4] Bernabucci U, Bani P, Ronchi B, Lacetera N, Nardone A (2010). Metabolic and hormonal acclimation to heat stress in domesticated ruminants. Animal..

[5] Stpierre NR, Cobanov B, Schnitkey G (2003). Economic losses from heat stress by US livestock Industries1. J Dairy Sci.

[6] Collier RJ, Eley RM, Sharma AK, Pereira RM, Buffington DE. Shade management in subtropical environment for milk yield and composition in Holstein and Jersey cows. J Dairy Sci. 1981;64(5):844–9.

[7] Linvill DE, Pardue FE. Heat stress and milk production in the South Carolina coastal plains. J Dairy Sci. 1992;75(9):2598. 10.3168/jds.S0022-0302(92)78022-9 1452860

[8] Mader TL, Davis MS, Brown-Brandl T (2006). Environmental factors influencing heat stress in feedlot cattle. J Anim Sci.

[9] West JW (2003). Effects of heat-stress on production in dairy cattle. J Dairy Sci.

[10] Colditz PJ, Kellaway RC. The effect of diet and heat stress on feed intake, growth, and nitrogen metabolism in Friesian, F_1_ Brahman × Friesian, and Brahman heifers. Aust J Agric Res. 1972;23(4):717–25.

[11] Stott GH, Wiersma F (1976). Influence of environment on passive immunity in calves. J Dairy Sci.

[12] Beede DK, Collier RJ (1986). Potential nutritional strategies for intensively managed cattle during thermal stress. J Anim Sci.

[13] Thom EC (1959). The discomfort index. Weatherwise..

[14] Dikmen S, Hansen P (2009). Is the temperature-humidity index the best indicator of heat stress in lactating dairy cows in a subtropical environment?. J Dairy Sci.

[15] Segnalini M, Bernabucci U, Vitali A, Nardone A, Lacetera N (2013). Temperature humidity index scenarios in the Mediterranean basin. Int J Biometeorol.

[16] Polsky L, von Keyserlingk MA (2017). Invited review: effects of heat stress on dairy cattle welfare. J Dairy Sci.

[17] Place NT, Heinrichs AJ, Erb HN (1998). The effects of disease, management, and nutrition on average daily gain of dairy heifers from birth to four months. J Dairy Sci.

[18] Rauba J, Heins BJ, Chesterjones H, Diaz HL, Ziegler D, Linn JG (2019). Relationships between protein and energy consumed from milk replacer and starter and calf growth and first-lactation production of Holstein dairy cows. J Dairy Sci.

[19] Broucek J, Kisac P, Uhrincat M (2009). Effect of hot temperatures on the hematological parameters, health and performance of calves. Int J Biometeorol.

[20] Baccari F, Johnson HD, Hahn GL (1983). Environmental heat effects on growth, plasma T3, and postheat compensatory effects on Holstein calves. Proc Soc Exp Biol Med.

[21] Nonaka I, Takusari N, Tajima K, Suzuki T, Higuchi K, Kurihara M (2008). Effects of high environmental temperatures on physiological and nutritional status of prepubertal Holstein heifers. Livest Sci.

[22] Purwanto BP, Nakamasu F, Yamamoto S (1993). Effect of environmental temperatures on heat production in dairy heifers differing in feed intake level. Asian Australas J Anim Sci.

[23] Sartori R, Sartor-Bergfelt R, Mertens SA, Guenther JN, Parrish JJ, Wiltbank MC (2002). Fertilization and early embryonic development in heifers and lactating cows in summer and lactating and dry cows in winter. J Dairy Sci.

[24] Collier RJ, Renquist BJ, Xiao Y (2017). A 100-year review: stress physiology including heat stress. J Dairy Sci.

[25] Pandey P, Hooda OK, Kumar S (2017). Impact of heat stress and hypercapnia on physiological, hematological, and behavioral profile of Tharparkar and Karan fries heifers. Vet World..

[26] Allen JD, Hall LW, Collier RJ, Smith JF (2015). Effect of core body temperature, time of day, and climate conditions on behavioral patterns of lactating dairy cows experiencing mild to moderate heat stress. J Dairy Sci.

[27] Hooda O, Upadhyay R (2015). Growth rate, hormonal and physiological responses of kids subjected to thermal and exercise stress. J Environ Res Dev.

[28] Benjamin M (1981). Fluid and electrolytes.

[29] Schneider PL, Beede DK, Wilcox CJ, Collier RJ (1984). Influence of dietary sodium and potassium bicarbonate and total potassium on heat-stressed lactating dairy cows. J Dairy Sci.

[30] Schneider PL, Beede DK, Wilcox CJ (1988). Nycterohemeral patterns of acid-base status, mineral concentrations and digestive function of lactating cows in natural or chamber heat stress environments. J Anim Sci.

[31] Neuwirth JG, Norton JK, Rawlings CA, Thompson FN, Ware GO (1979). Physiologic responses of dairy calves to environmental heat stress. Int J Biometeorol.

[32] O’Brien MD, Rhoads RP, Sanders SR, Duff GC, Baumgard LH (2010). Metabolic adaptations to heat stress in growing cattle. Domest Anim Endocrinol.

[33] Bernabucci U, Ronchi B, Lacetera N, Nardone A (2002). Markers of oxidative status in plasma and erythrocytes of transition dairy cows during hot season. J Dairy Sci.

[34] Belhadj Slimen I, Najar T, Ghram A, Abdrrabba M (2016). Heat stress effects on livestock: molecular, cellular and metabolic aspects, a review. J Anim Physiol Anim Nutr.

[35] Cole L, Skrzypek M, Sanders S, Waldron M, Baumgard L, Rhoads R (2011). Effects of heat stress on skeletal muscle insulin responsiveness in lactating Holstein cows. J Dairy Sci.

[36] Baumgard LH, Rhoads RP. Effects of heat stress on postabsorptive metabolism and energetics. Annu Rev Anim Biosci. 2013;1(1):311–37. 10.1146/annurev-animal-031412-103644 25387022

[37] Vernon RG (1992). Effects of diet on lipolysis and its regulation. Proc Nutr Soc.

[38] Magdub A, Johnson HD, Belyea RL. Effect of environmental heat and dietary fiber on thyroid physiology of lactating cows 1. J Dairy Sci. 1982;65(12):2323–31. 10.3168/jds.S0022-0302(82)82504-6 6298292

[39] Bernabucci U, Bani P, Ronchi B, Lacetera N, Nardone A (1999). Influence of short- and long-term exposure to a hot environment on rumen passage rate and diet digestibility by Friesian heifers. J Dairy Sci.

[40] Yadav B, Singh G, Verma A, Dutta N, Sejian V (2013). Impact of heat stress on rumen functions. Vet World..

[41] Tajima K, Nonaka I, Higuchi K, Takusari N, Kurihara M, Takenaka A (2007). Influence of high temperature and humidity on rumen bacterial diversity in Holstein heifers. Anaerobe..

[42] McDowell R, Moody E, Van Soest P, Lehmann R, Ford G (1969). Effect of heat stress on energy and water utilization of lactating cows. J Dairy Sci.

[43] Rogers J, Davis C (1982). Rumen volatile fatty acid production and nutrient utilization in steers fed a diet supplemented with sodium bicarbonate and monensin. J Dairy Sci.

[44] Silanikove N (1992). Effects of water scarcity and hot environment on appetite and digestion in ruminants: a review. Livest Prod Sci.

[45] Kocherginskaya SA, Aminov RI, White BA (2001). Analysis of the rumen bacterial diversity under two different diet conditions using denaturing gradient gel electrophoresis, random sequencing, and statistical ecology approaches. Anaerobe..

[46] Tajima K, Aminov R, Nagamine T, Matsui H, Nakamura M, Benno Y (2001). Diet-dependent shifts in the bacterial population of the rumen revealed with real-time PCR. Appl Environ Microbiol.

[47] Uyeno Y, Sekiguchi Y, Tajima K, Takenaka A, Kurihara M, Kamagata Y (2010). An rRNA-based analysis for evaluating the effect of heat stress on the rumen microbial composition of Holstein heifers. Anaerobe..

[48] Russell JB, Rychlik JL (2001). Factors that alter rumen microbial ecology. Science..

[49] Dehority BA (2003). Rumen microbiology.

[50] Weldy J, McDowell R, Van Soest P, Bond J (1964). Influence of heat stress on rumen acid levels and some blood constituents in cattle. J Anim Sci.

[51] Gangwar PC, Branton C, Evans DL (1965). Reproductive and physiological responses of Holstein heifers to controlled and natural climatic conditions 1. J Dairy Sci.

[52] Bolocan E (2009). Effects of heat stress on sexual behavior in heifers. Sci Papers Anim Sci Biotechnol.

[53] De Rensis F, Scaramuzzi RJ (2003). Heat stress and seasonal effects on reproduction in the dairy cow—a review. Theriogenology..

[54] Bilby TR, Baumgard LH, Collier RJ, Zimbelman RB, Rhoads ML. Heat stress effects on fertility: Consequences and possible solutions. The Proceedings of the 2008 South Western Nutritional Conference. 2008.

[55] Badinga L, Thatcher W, Diaz T, Drost M, Wolfenson D (1993). Effect of environmental heat stress on follicular development and steroidogenesis in lactating Holstein cows. Theriogenology..

[56] Howell J, Fuquay J, Smith A (1994). Corpus luteum growth and function in lactating Holstein cows during spring and summer. J Dairy Sci.

[57] Wilson S, Kirby C, Koenigsfeld A, Keisler D, Lucy M (1998). Effects of controlled heat stress on ovarian function of dairy cattle. 2. Heifers. J Dairy Sci.

[58] Hansen P, Drost M, Rivera R, Paula-Lopes F, Al-Katanani Y, Krininger C (2001). Adverse impact of heat stress on embryo production: causes and strategies for mitigation. Theriogenology..

[59] Roth Z, Meidan R, Shaham-Albalancy A, Braw-Tal R, Wolfenson D (2001). Delayed effect of heat stress on steroid production in medium-sized and preovulatory bovine follicles. Reprod Cambridge.

[60] Roman-Ponce H, Thatche WW, Caton D, Barron DH, Wilcox CJ (1978). Thermal stress effects on uterine blood flow in dairy cows. J Anim Sci.

[61] Gwazdauskas F, Thatcher W, Kiddy C, Paape M, Wilcox C (1981). Hormonal patterns during heat stress following PGF2α-tham salt induced luteal regression in heifers. Theriogenology..

[62] Hein KG, Allrich RD (1992). Influence of exogenous adrenocorticotropic hormone on estrous behavior in cattle. J Anim Sci.

[63] Khodaei-Motlagh M, Shahneh AZ, Masoumi R, Derensis F (2011). Alterations in reproductive hormones during heat stress in dairy cattle. Afr J Biotechnol.

[64] Putney DJ, Drost M, Thatcher WW (1988). Embryonic development in superovulated dairy cattle exposed to elevated ambient temperatures between days 1 to 7 post insemination. Theriogenology..

[65] Tao S, Dahl G (2013). Invited review: heat stress effects during late gestation on dry cows and their calves. J Dairy Sci.

[66] Ealy AD, Drost M, Hansen PJ (1993). Developmental changes in embryonic resistance to adverse effects of maternal heat stress in cows 1. J Dairy Sci.

[67] Edwards JL, Hansen PJ (1996). Elevated temperature increases heat shock protein 70 synthesis in bovine two-cell embryos and compromises function of maturing oocytes. Biol Reprod.

[68] Wolfenson D, Roth Z, Meidan R (2000). Impaired reproduction in heat-stressed cattle: basic and applied aspects. Anim Reprod Sci.

[69] Fear JM, Hansen PJ (2011). Developmental changes in expression of genes involved in regulation of apoptosis in the bovine preimplantation embryo. Biol Reprod.

[70] Hansen P (2007). Exploitation of genetic and physiological determinants of embryonic resistance to elevated temperature to improve embryonic survival in dairy cattle during heat stress. Theriogenology..

[71] Bauman DE, Currie WB (1980). Partitioning of nutrients during pregnancy and lactation: a review of mechanisms involving homeostasis and homeorhesis. J Dairy Sci.

[72] Reynolds LP, Caton JS, Redmer DA, Grazul-Bilska AT, Vonnahme KA, Borowicz PP (2006). Evidence for altered placental blood flow and vascularity in compromised pregnancies. J Physiol.

[73] Collier RJ, Doelger S, Head H, Thatcher W, Wilcox C (1982). Effects of heat stress during pregnancy on maternal hormone concentrations, calf birth weight and postpartum milk yield of Holstein cows. J Anim Sci.

[74] Thompson I, Tao S, Branen J, Ealy A, Dahl G (2013). Environmental regulation of pregnancy-specific protein B concentrations during late pregnancy in dairy cattle. J Anim Sci.

[75] Bell AW, Ehrhardt RA (2002). Regulation of placental nutrient transport and implications for fetal growth. Nutr Res Rev.

[76] Wu G, Bazer F, Wallace J, Spencer T (2006). Board-invited review: intrauterine growth retardation: implications for the animal sciences. J Anim Sci.

[77] Tao S, Monteiro A, Thompson I, Hayen M, Dahl G (2012). Effect of late-gestation maternal heat stress on growth and immune function of dairy calves. J Dairy Sci.

[78] Monteiro A, Guo J-R, Weng X-S, Ahmed B, Hayen M, Dahl G (2016). Effect of maternal heat stress during the dry period on growth and metabolism of calves. J Dairy Sci.

[79] Laporta J, Fabris T, Skibiel A, Powell J, Hayen M, Horvath K (2017). In utero exposure to heat stress during late gestation has prolonged effects on the activity patterns and growth of dairy calves. J Dairy Sci.

[80] Nardone A, Lacetera N, Bernabucci U, Ronchi B (1997). Composition of colostrum from dairy heifers exposed to high air temperatures during late pregnancy and the early postpartum period. J Dairy Sci.

[81] Ahmed B, Younas U, Asar T, Dikman S, Hansen P, Dahl G (2015). Cows exposed to heat stress in utero exhibit improved thermal tolerance. J Dairy Sci.

[82] Monteiro A, Tao S, Thompson I, Dahl G (2016). In utero heat stress decreases calf survival and performance through the first lactation. J Dairy Sci.

[83] Hansen P, Areéchiga C (1999). Strategies for managing reproduction in the heat-stressed dairy cow. J Anim Sci.

[84] Dikmen S, Alava E, Pontes E, Fear J, Dikmen B, Olson T (2008). Differences in thermoregulatory ability between slick-haired and wild-type lactating Holstein cows in response to acute heat stress. J Dairy Sci.

[85] Mishra SR, Palai TK. Importance of heat shock protein 70 in livestock at cellular level. J Mol Pathophysiol. 2014;3(2):30–2.

[86] Das R, Sailo L, Verma N, Bharti P, Saikia J (2016). Impact of heat stress on health and performance of dairy animals: a review. Vet World.

[87] Putney D, Drost M, Thatcher W (1989). Influence of summer heat stress on pregnancy rates of lactating dairy cattle following embryo transfer or artificial insemination. Theriogenology..

[88] Ambrose J, Drost M, Monson R, Rutledge J, Leibfried-Rutledge M, Thatcher M-J (1999). Efficacy of timed embryo transfer with fresh and frozen in vitro produced embryos to increase pregnancy rates in heat-stressed dairy cattle. J Dairy Sci.

[89] Jousan F, Hansen P (2004). Insulin-like growth factor-I as a survival factor for the bovine preimplantation embryo exposed to heat shock. Biol Reprod.

[90] Jousan FD, Hansen PJ (2007). Insulin-like growth factor-I promotes resistance of bovine preimplantation embryos to heat shock through actions independent of its anti-apoptotic actions requiring PI3K signaling. Mol Reprod Dev.

[91] Pejman A, Shahryar A (2012). Heat stress in dairy cows (a review). Res Zool.

[92] Gebremedhin KG, Hillman PE, Lee CN, Collier RJ, Willard ST, Arthington JD (2008). Sweating rates of dairy cows and beef heifers in hot conditions. Trans ASABE.

[93] Marai I, Habeeb A, Daader A, Yousef H (1995). Effects of Egyptian subtropical summer conditions and the heat-stress alleviation technique of water spray and a diaphoretic on the growth and physiological functions of Friesian calves. J Arid Environ.

[94] Hill T, Bateman H, Aldrich J, Schlotterbeck R (2011). Comparisons of housing, bedding, and cooling options for dairy calves. J Dairy Sci.

[95] Moghaddam A, Karimi I, Pooyanmehr M (2009). Effects of short-term cooling on pregnancy rate of dairy heifers under summer heat stress. Vet Res Commun.

[96] Bond T, Kelly C (1955). The globe thermometer in agricultural research. Agric Eng.

[97] Marcillac-Embertson N, Robinson P, Fadel J, Mitloehner FM (2009). Effects of shade and sprinklers on performance, behavior, physiology, and the environment of heifers. J Dairy Sci.

[98] Kurihara M (1996). Energy requirements and feed of dairy cows under high temperature conditions. Jpn Agric Res Q.

[99] West JW (1999). Nutritional strategies for managing the heat-stressed dairy cow. J Anim Sci.

[100] Escobosa A, Coppock CE, Rowe Jr LD, Jenkins WL, Gates CE. Effects of dietary sodium bicarbonate and calcium chloride on physiological responses of lactating dairy cows in hot weather. J Dairy Sci. 1984;67(3):574–84. 10.3168/jds.S0022-0302(84)81341-7 6325517

[101] Escobosa A, Coppock CE, Rowe Jr LD, Jenkins WL, Gates CE. Effects of dietary sodium bicarbonate and calcium chloride on physiological responses oflactating dairy cows in hot weather. J Dairy Sci. 1984;67(3):574–84. 10.3168/jds.S0022-0302(84)81341-7 6325517

